# Synthesis of ketones from biomass-derived feedstock

**DOI:** 10.1038/ncomms14190

**Published:** 2017-01-31

**Authors:** Qinglei Meng, Minqiang Hou, Huizhen Liu, Jinliang Song, Buxing Han

**Affiliations:** 1 Beijing National Laboratory for Molecular Sciences, CAS Key Laboratory of Colloid and Interface and Thermodynamics, Institute of Chemistry, Chinese Academy of Sciences, Beijing 100190, China; 2 School of Chemistry and Chemical Engineering, University of Chinese Academy of Sciences, Beijing 100049, China

## Abstract

Cyclohexanone and its derivatives are very important chemicals, which are currently produced mainly by oxidation of cyclohexane or alkylcyclohexane, hydrogenation of phenols, and alkylation of cyclohexanone. Here we report that bromide salt-modified Pd/C in H_2_O/CH_2_Cl_2_ can efficiently catalyse the transformation of aromatic ethers, which can be derived from biomass, to cyclohexanone and its derivatives via hydrogenation and hydrolysis processes. The yield of cyclohexanone from anisole can reach 96%, and the yields of cyclohexanone derivatives produced from the aromatic ethers, which can be extracted from plants or derived from lignin, are also satisfactory. Detailed study shows that the Pd, bromide salt and H_2_O/CH_2_Cl_2_ work cooperatively to promote the desired reaction and inhibit the side reaction. Thus high yields of desired products can be obtained. This work opens the way for production of ketones from aromatic ethers that can be derived from biomass.

## Abstract (web summary)

Conversion of biomass-derived chemicals to industrially relevant products can allow sustainable production of organic compounds. Here the authors report that aromatic ethers, which can be derived for biomass, can be converted into various cyclohexanones via a bromide salt-modified palladium catalyst.

Cyclohexanone is a key raw material for producing nylon 6 and nylon 66 and for synthesis of other chemicals^1^. The industrial production of cyclohexanone typically involves either the oxidation of cyclohexane^2^ or the hydrogenation of phenol^3^. Cyclohexanone derivatives are also useful chemicals or intermediates to produce valuable chemicals. For example, methylcyclohexanone is used for preparing cyclohexanone imines, which can be followed by dehydrogenative aromatization to generate arylamines that are the core structures of various functional molecules with biological activities and optoelectronic properties relevant to pharmaceuticals and materials science, respectively^4^. 4-Propylcyclohexanone is used for the synthesis of chiral tacrine analogues which show important pharmacological activities^5^. The conventional synthesis of cyclohexanone derivatives typically involves either the oxidation of alkylcyclohexane^6^ or the alkylation of cyclohexanone^7^.

Utilization of biomass as raw materials to produce useful chemical compounds can liberate us from the reliance on fossil resource, and can also be considered as recycling of CO_2_ by combination of photosynthesis and chemical methods^8^. Lignocellulosic biomass is abundant renewable carbon resource, and lignin is the main constituent of lignocellulosic biomass^9^,^10^. However, lignin has nearly not been used in industry, especially for producing valuable compounds^11^. It is well known that lignin is rich in aromatic ether segments and aromatic ether bonds widely exist in the structure of lignin^12^. In recent years, much attention has been paid to the depolymerization of lignin, and various useful products and platform molecules have been obtained, such as liquid fuels^13^,^14^, alcohols^10^,^15^, and aromatic ethers^15^,^16^,^17^,^18^,^19^,^20^. It can be expected that more feasible methods will be developed to produce various aromatic ethers from lignin. Moreover, aromatic ethers, such as anethole, *trans*-anethole, exist widely in plants and can be directly extracted^21^,^22^,^23^. The representative aromatic ethers depicted in Fig. 1 illustrates that the natural aromatic carbon-oxygen (C_aromatic_-O) bonds may directly provide structural integrity for the ketone-type structure of cyclohexanone by means of catalytic valorization^9^. Runnebaum *et al*.^24^ performed the conversion of anisole in the presence of H_2_ on Pt/Al_2_O_3_ catalyst at 300 °C, generating cyclohexanone with a selectivity of 3% and trace amounts of 2-methylcyclohexanone. Hubert *et al*.^25^ carried out the hydrogenation of anisole using THEA16Cl-stabilized Rh (0) nanoparticles as catalyst, and the yield of cyclohexanone reached 22%.

It can be anticipated that cyclohexanone and its derivatives can be produced in sustainable way if we can develop efficient protocols to transform aromatic ethers into these important chemicals, as shown in Fig. 1. However, achieving high yield is a great challenge because aromatic ethers tend to completely hydrogenated to alkyl ethers, and ketone products can be further hydrogenated to cyclohexanol and its derivatives under the reaction conditions. Up to now, only the transformation of anisole to cyclohexanone has been reported, and the highest yield was 22%. In this work, we developed a route for the production of cyclohexanone and its derivatives from aromatic ethers. It was discovered that bromide salt modified Pd/C (m-Pd/C) catalyst in H_2_O/CH_2_Cl_2_ medium was highly efficient for the transformation of aromatic ethers to ketones, and the yield of cyclohexanone could reach 96% for the conversion of anisole to cyclohexanone. This catalytic system could also be applied to transform other aromatic ethers to ketones with high yields. This work opens the way for sustainable producing ketones from bio-based feedstocks.

## Results

### Catalytic system screening

We first used anisole to study the conversion of aromatic ethers to cyclohexanone and its derivatives because it has only one methoxy group that simplifies the study of the reaction. Commercial Pd/C catalyst (Supplementary Fig. 1) was used because it is a very commonly used catalyst for hydrogenation reactions. Table 1 presents the results of the conversion of anisole under different conditions. The reaction in different solvents was studied using Pd/C as the catalyst. Cyclohexyl methyl ether (3) was the only product in organic solvents (Table 1, entries 1–4). Cyclohexanone (1), cyclohexanol (2), and cyclohexyl methyl ether (3) were produced in water, and the selectivity to cyclohexanone (1) was 13.0% at 99.8% conversion of anisole (Table 1, entry 5), indicating that water was essential for the generation of cyclohexanone. As expected, the reaction did not occur without catalyst and/or H_2_ (Table 1, entry 6). We also performed the reaction using Pt/C, Ru/C and Rh/C catalysts in water (Table 1, entries 7–9). Under the similar conversion of anisole, only 1.5% yield of cyclohexanone was obtained over Pt/C catalyst, while selectivity of the by-product cyclohexyl methyl ether (3) was higher than 80% (Table 1, entry 7). Especially, in the presence of Ru/C and Rh/C catalysts, no cyclohexanone was detected (Table 1, entries 8–9). The results above indicate that Pd/C is the best catalyst among the catalysts checked for the transformation of anisole to cyclohexanone. Considering the solubility of the solvent, some binary solvents containing water and organic solvent were used for the reaction, and H_2_O/CH_2_Cl_2_ exhibited the best performance for producing cyclohexanone (Table 1, entries 10–12), and the yield of cyclohexanone reached 67.7%. It has been reported that small amount of halide anions could improve the selectivity of some reactions over Pd catalyst^26^,^27^. Therefore, to further improve the yield of cyclohexanone, the effect of some halide salts on the reaction was studied (Table 1, entries 13–18). Similarly, cyclohexanone was not formed without water (Table 1, entry 13). All the salts with Br^−^ (KBr and NaBr) could enhance the selectivity to cyclohexanone effectively in water or H_2_O/CH_2_Cl_2_ (Table 1, entries 14–16), and particularly high selectivity could be obtained when H_2_O/CH_2_Cl_2_ was used as the solvent. However, KCl could not improve the selectivity considerably (Table 1, entry 17). In addition, KI completely prohibited the conversion of anisole (Table 1, entry 18), which is consistent with the argument that I^−^ can deactivate Pd catalyst seriously^28^,^29^.

To study the reason for the high selectivity of the desired product, some control experiments (Supplementary Fig. 2) were conducted. Supplementary Fig. 2a demonstrates the dependence of yields of the products on reaction time in neat water over Pd/C. Cyclohexanone and cyclohexyl methyl ether are the main products in 1.5 h. However, the selectivity of cyclohexanone decreased quickly after 1.5 h, accompanying the increase of the selectivity of cyclohexanol because Pd can catalyse the hydrogenation of cyclohexanone to cyclohexanol^3^. To explain this result, we calculated the adsorption energy of anisole and cyclohexanone on Pd (111) surface by density function theory (DFT) method^30^,^31^,^32^, and the values are −0.74 and −0.26 eV, respectively (Supplementary Fig. 3). This suggests that anisole is more easily adsorbed on the surface of the catalyst and occupy the active site than cyclohexanone. So the anisole in the reaction system can prevent the adsorption of cyclohexanone on the catalyst and inhibit its hydrogenation to cyclohexanol, especially at higher anisole concentration. Thus, the DFT calculation can explain the phenomenon why the selectivity to cyclohexanone decreased quickly at higher anisole conversion (Supplementary Fig. 2a). Supplementary Fig. 2b shows the results of the reaction in H_2_O/CH_2_Cl_2_. It can be known by comparing Supplementary Fig. 2a,b that selectivity with cyclohexyl methyl ether in H_2_O/CH_2_Cl_2_ was lower than that in neat water. In other words, H_2_O/CH_2_Cl_2_ solvent is not favourable to the generation of the by-product. Supplementary Fig. 2c shows the reaction results in KBr aqueous solution without CH_2_Cl_2_. It can be known by comparing Supplementary Fig. 2a,c that KBr could suppress the hydrogenation of cyclohexanone with cyclohexanol effectively, and could also reduce the selectivity to cyclohexyl methyl ether notably. To further confirm the effect of KBr, we conducted the hydrogenation of cyclohexanone in H_2_O, H_2_O/CH_2_Cl_2_, aqueous solution of KBr and H_2_O/CH_2_Cl_2_+KBr system under the same reaction conditions, and the results are presented in Supplementary Fig. 2d. Obviously, KBr was highly effective to prevent the hydrogenation of cyclohexanone, especially in H_2_O/CH_2_Cl_2_. From the results in Supplementary Fig. 2, we can conclude that H_2_O/CH_2_Cl_2_ can inhibit the formation of cyclohexyl methyl ether, and KBr can prevent hydrogenation of cyclohexanone and suppress generation of cyclohexyl methyl ether. Therefore, the selectivity of cyclohexanone could be outstandingly high in H_2_O/CH_2_Cl_2_ with KBr. It is well-known that Br^−^ can be adsorbed on Pd catalyst^28^, which may be the main reason for the bromide salts to improve the selectivity of the reaction effectively. We carried out some experiments to verify this argument. In the experiments, the Pd/C catalyst was dispersed in KBr aqueous solution, and the mixture was stirred for 1 h. Then, m-Pd/C was obtained after filtration, washing, and drying. The adsorption of Br^−^ in the m-Pd/C was confirmed by the XPS (Supplementary Figs 4 and 5). The m-Pd/C was used to catalyse the reaction without adding additional KBr. And the selectivity to cyclohexanone could reach 96% (Table 1, entries 19 and 20), which was even higher than that when Pd/C was used with addition of KBr. This is understandable considering that in the latter case more by-product was formed at beginning because the adsorption of Br^−^ needs some time. SiO_2_, CaCl_2_, K_2_CO_3_ and MgSO_4_ are the impurities of biomass-derived streams^33^. Their effect on the reaction were also investigated (Table 1, entries 21–24). It was demonstrated that the effect of the impurities on the catalytic performance of the catalytic system was not considerable. In the following, we study the effects of various conditions on the reaction in H_2_O/CH_2_Cl_2_ over the Br- modified catalyst because they showed excellent performance.

### Optimization of reaction conditions

**Figure 2:**
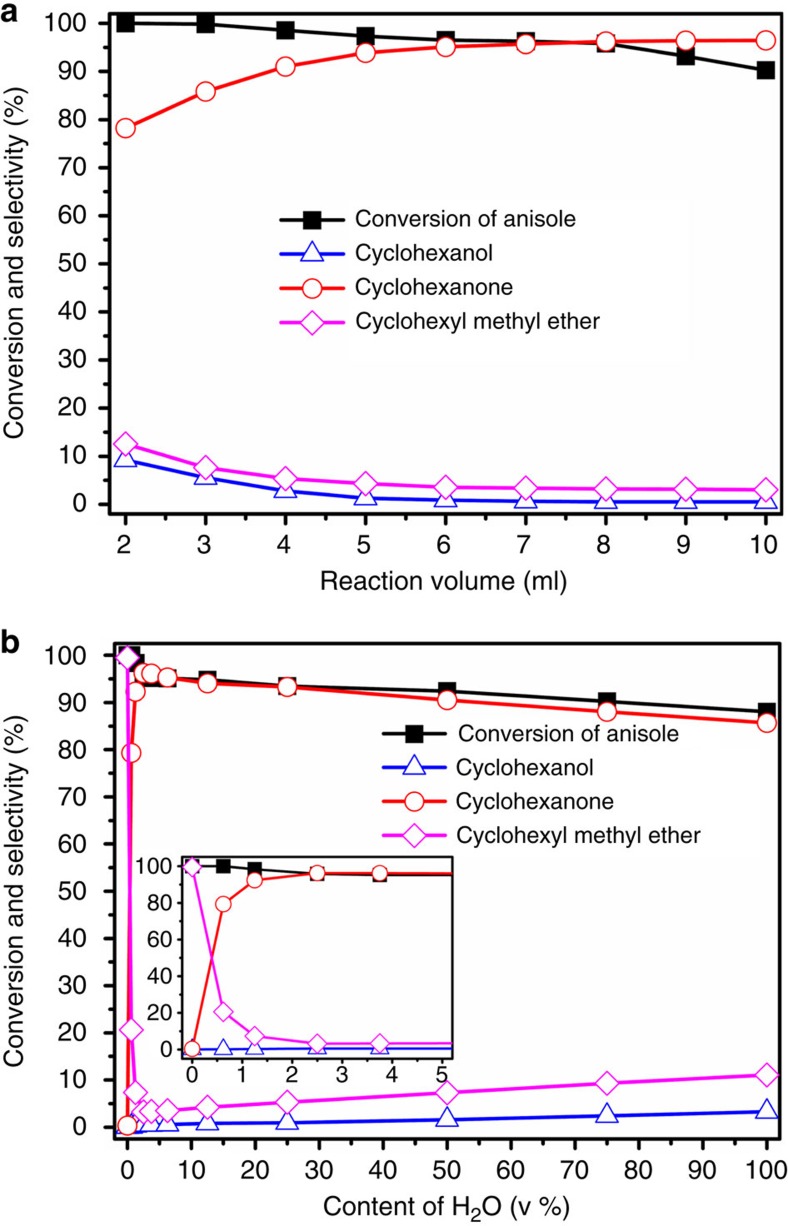
Effects of solvent volume and content of water. Effects of solvent volume with a water amount of 0.2 ml (**a**) and content of H_2_O at total solvent volume of 8.0 ml (**b**) on the conversion and product distribution of the reaction over m-Pd/C catalyst. Reaction conditions: anisole (1.5 mmol), 5 wt% m-Pd/C (0.03 g, 1.41 × 10^−2^ mmol Pd), 90 °C, 2.5 h, 2 MPa.

The effects of amount of solvent used and H_2_O content in H_2_O/CH_2_Cl_2_ on the catalytic reaction over the m-Pd/C are shown in Fig. 2. The selectivity of cyclohexanone increased and that of cyclohexyl methyl ether decreased with the increase of the amount of solvent (Fig. 2a). However, the conversion of anisole decreased with increasing the volume of the solvent, which is understandable based on rate equation. Figure 2b demonstrates effect of H_2_O content on the catalytic reaction. The conversion of anisole decreased continuously with increase of H_2_O content in H_2_O/CH_2_Cl_2_. This is understandable because the conversion of anisole in water was slower than in CH_2_Cl_2_, as can be known from Table 1 (entries 1 and 5). The selectivity of cyclohexanone increased sharply with increasing H_2_O content at the beginning from 0 to 2.5 v%, and then decreased gradually with further increase of H_2_O content from 2.5 to 100 v%. The trend of the selectivity of cyclohexyl methyl ether was opposite to that of cyclohexanone. The selectivity of cyclohexanol increased slowly with increasing content of H_2_O in the solvent. The results indicated that water is essential for the production of cyclohexanone. However, more water would promote the side reaction of the transformation of anisole to cyclohexyl methyl ether and the further hydrogenation of cyclohexanone to cyclohexanol.

The effects of temperature, H_2_ pressure and reaction time on the conversion and product distribution of the reaction over the m-Pd/C in H_2_O/CH_2_Cl_2_ are shown in Supplementary Fig. 6a–c. The results show that the optimized temperature and H_2_ pressure were 90 °C and 2 MPa, respectively. We also studied the reusability of the m-Pd/C. After reaction, the liquid was removed and the m-Pd/C catalyst was reused directly after washing and drying. The conversion of anisole and selectivity to cyclohexanone did not change notably after the catalyst was reused five times (Supplementary Fig. 6d). The m-Pd/C catalysts before and after using five times was also characterized by XPS, X-ray diffraction and TEM techniques. XPS spectra (Supplementary Figs 4 and 5) demonstrate that the Br^−^ adsorbed on the Pd surface was stable in the reaction process. The X-ray diffraction patterns (Supplementary Fig. 7) and TEM images (Supplementary Figs 1 and 8) indicate that the crystalline and particle size of the Pd nanoparticles did not change in the reaction. To further check the stability of the catalyst, the m-Pd/C catalyst in H_2_O/CH_2_Cl_2_ was stirred at 120 °C for 40 h (equivalent to the time for eight reaction cycles) in the absence of anisole. Then the catalyst was used for the reaction at the condition of entry 19 of Table 1. The conversion of anisole and selectivity to cyclohexanone were 95.0% and 96.6% respectively, which were nearly the same as that obtained using the virgin m-Pd/C catalyst, which further indicates the excellent stability of the m-Pd/C catalyst.

### Reaction mechanism

**Figure 3:**
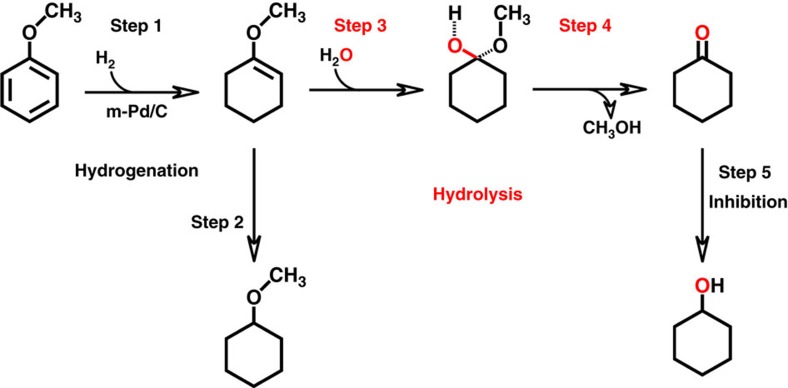
Proposed reaction pathway for the transformation of anisole over the m-Pd/C catalyst in H_2_O/CH_2_Cl_2_ medium. The benzene ring of anisole is first partially hydrogenated to form 1-methoxycyclohexene on Pd catalyst in Step 1, which can be further hydrogenated to cyclohexyl methyl ether (Step 2) or hydrolysed to cyclohexanone (Steps 3–4). Cyclohexanone can then be further hydrogenated to form cyclohexanol (Step 5). The m-Pd/C and H_2_O/CH_2_Cl_2_ catalytic system promoted the production of cyclohexanone and inhibited the production of cyclohexyl methyl ether and cyclohexanol.

It is well known that Pd can activate H_2_ (ref. 34) and catalyse the partial hydrogenation of the benzene ring of phenol to an enol^3^,^35^. To study the reaction mechanism, identification of intermediate in the transformation of anisole was carried out in this work, and 1-methoxycyclohexene was detected (Supplementary Fig. 9). On the basis of the experimental results in this work and the related knowledge in the literature, we propose the possible pathway of the reaction, which is shown schematically in Fig. 3. The benzene ring of anisole is first partially hydrogenated to form 1-methoxycyclohexene on Pd catalyst in Step 1, which can be further hydrogenated to cyclohexyl methyl ether (Step 2)^36^ or hydrolysed to cyclohexanone (Steps 3–4)^37^,^38^. Cyclohexanone can then be further hydrogenated to form cyclohexanol (Step 5). The experimental results support the proposed pathway. For example, cyclohexanone cannot be formed without water (Table 1), and the yield of methanol is similar to that of the total yield of the cyclohexanone and cyclohexanol (Supplementary Table 1), and no gaseous product was produced in the anisole transformation (Supplementary Fig. 10). All the results support the hydrolysis route (Steps 3–4). Besides, the isotope tracing experiment using H_2_^18^O indicated that only C=^18^O bond existed in cyclohexanone (Supplementary Fig. 11), further supporting the proposed pathway. Furthermore, we conducted the transformation of 1-methoxycyclohexene (Supplementary Fig. 12). It was shown that 1-methoxycyclohexene could be converted rapidly under reaction conditions and the product distribution was the same as that of anisole transformation.

### Substrate scope

We also studied the catalytic performance of the m-Pd/C in H_2_O/CH_2_Cl_2_ for the conversion of other aromatic ethers to cyclohexanone or its corresponding derivatives. Table 2 gives the optimized reaction conditions and the yields of corresponding desired products for various substrates. In general, the catalytic system was more efficient for anisole derivatives with electron-donating substituents than those with electron-withdrawing substituents (Table 2, entries 1–14). 4-Methyl anisole (Table 2, entry 1) and 2-methyl anisole (Table 2, entry 2) could be converted to 4-methyl cyclohexanone and 2-methyl cyclohexanone with the yields of 80.1% and 90.3%, respectively. 4-Ethyl anisole (Table 2, entry 3) and 2-ethyl anisole (Table 2, entry 4) could be converted to 4-ethyl cyclohexanone and 2-ethyl cyclohexanone and the yields were 81.5% and 88.1%, respectively. Moreover, 2-methoxylphenol, 2-methoxy-4-methylphenol, and 4-ethyl-2-methoxyphenol can be produced from depolymerization of lignin^15^. They could be converted to the corresponding ketones in this catalytic system, and the overall yields of the ketones were 64.8% (Table 2, entry 5), 74.2% (Table 2, entry 6) and 60.2% (Table 2, entry 7), respectively. Anethole and *trans*-anethole exist widely in anise, fennel and tarragon, which can be extracted^21^,^22^,^23^. Anethole (Table 2, entry 8) and *trans*-anethole (Table 2, entry 9) could be converted into to 4-propylcyclohexanone with yields of 77.3% and 71.8%, respectively. However, anisole with electron-withdrawing substituents such as ester group, trifluoromethyl group and formyl group afforded low yield of ketone product (Table 2, entries 10–14). The yield of 3-oxo-cyclohexanecarboxylic acid methyl ester was 41.6% (Table 2, entry 10). When ester group was on para-position, the yield of desired product decreased to 35.1% (Table 2, entry 11). The yield of 3-(trifluoromethyl) cyclohexanone was 15.1% and the yield of 4-(trifluoromethyl) cyclohexanone was 0% (Table 2, entries 12 and 13). Formyl substitute afforded 0% yield of the desired product (Table 2, entry 14). In addition, the catalytic system could also catalyse the transformation of benzyl phenyl ether and diphenyl ether that represent typical ethers with α-O-4 and 4-O-5 linkages in lignin and are typical lignin-derived compounds^13^,^14^ to cyclohexanone, and the yields of cyclohexanone could reach 93.0% and 94.2%, respectively (Table 2, entries 15 and 16).

## Discussion

m-Pd/C in H_2_O/CH_2_Cl_2_ can catalyse the transformation of aromatic ethers to cyclohexanone or its corresponding derivatives via hydrogenation and hydrolysis processes, and high to moderate yield of the corresponding ketones can be obtained. In general, the catalytic system is more efficient for anisole derivatives with electron-donating substituents than those with electron-withdrawing substituents. Detailed study indicates that m-Pd/C and H_2_O/CH_2_Cl_2_ cooperatively promote the partial hydrogenation to vinyl ethers and their hydrolysis to ketones, and the bromide anions adsorbed on the surface of Pd catalyst inhibit the hydrogenation of ketones and suppress generation of byproduct. Thus, the selectivity to the desired ketone products can be very high. In addition, m-Pd/C can be reused at least five times without reducing the activity and selectivity notably, indicating that the catalytic system is very stable. Aromatic ethers can be extracted from some plants directly. In addition, study on depolymerization of lignin to produce low-molecular-weight compounds is receiving increasing attention in recent years, and more and more aromatic ethers may be produced from lignin in the future. We believe that the route reported in this work has great potential of application for producing ketones sustainably.

## Methods

### Materials

Anisole (99.0%), cyclohexanol (99.0%), 2-cyclohexylethanol (99.0%), biphenyl (99.0%), phenol (99.0%), cesium bromide (99.9%), diphenyl ether (99%), 2-methoxy-4-methylphenol (98%), 4-ethyl-2-methoxyphenol (98%), 4-ethylanisole (98%), 4-(trifluoromethyl)cyclohexanone (97%) and silicon oxide were purchased from Alfa Aesar. Guaiacol (99.0%), (E)-1-methoxy-4-(prop-1-en-1-yl), benzene (*trans*-anethol) (99.0%), 2-methylanisole (99.0%), 4-methylanisole (99.0%), 2-methylcyclohexanone (98.0%), 4-methylcyclohexanone (98.0%) and 3-methylcyclohexanone (97.0%) were purchased from Acros Organics. 1-Methoxy-4-(prop-1-en-1-yl) benzene (*cis*-anethol) (97.0%), 4-ethylcyclohexanone (95.0%), 4-hydroxycyclohexanone (99.0%) and 1-methoxy-4-(trifluoromethyl)benzene (95%) were purchased from Ark Pharm. 2-Ethylanisole (98%), methylcyclohexane (99.0%), ethylcyclohexane (98.0%), methoxycyclohexane (98.0%), benzyl phenyl ether (98.0%), 4-propylcyclohexanone (98.0%), 4-propylcyclohexanol (98.0%) and 2-methoxycyclohexanone (95.0%) were obtained from TCI (Shanghai) Development Co., Ltd. 4-Propylanisole (99.0%), 3-(trifluoromethyl)anisole (99%), 4-methoxybenzamide (97%), methyl 4-methoxybenzoate (99%), methyl 3-methoxybenzoate (98%), methyl 2-methoxybenzoate (97%), 2-methoxycarbonylcyclohexanone (90%) and H_2_^18^O (97 atom%^18^O) were purchased from J & K Scientific Ltd. Chloroform-D (D, 99.8% +3% v/v TMS) was purchased from Cambridge Isotope Laboratories, Inc.. Potassium bromide (99.0%), potassium chloride (99.5%), potassium iodide (99.0%), sodium bromide (A. R.), Magnesium sulfate anhydrous (98.0%), calcium chloride anhydrous (96.0%), potassium carbonate anhydrous (99.0%), *n*-hexane (A. R.), tetrahydrofuran (A. R.), 2-phenylethyl bromide (99.0%), toluene (99.5%) and ethylbenzene (98.5%) were obtained from Sinopharm Chemical Reagent Co., Ltd. Methanol (99.9%) ethanol (99.9%), ethyl acetate (99.5%), dichloromethane (99.5%), cyclohexanone (99.5%) and cyclohexane (99.5%) were obtained from the Beijing Chemical Company. 1-Methoxycyclohexene (a mixture of 50 mol% 1-methoxycyclohexene and 50 mol% cyclohexanone dimethylacetal) was purchased from Toronto Research Chemicals. Nitrogen (>99.99%) and hydrogen (>99.99%) were provided by Beijing Analytic Instrument Company.

### Catalysts and characterization

Pd/C (5 wt% Pd), Pt/C (5 wt% Pt), Rh/C (5 wt% Rh) were purchased from Alfa Aesar. Ru/C (5 wt% Ru) was obtained from TCI (Shanghai) Development Co., Ltd. m-Pd/C catalyst was prepared in a 50 ml Teflon-lined stainless-steel reactor equipped with a stirrer. In a typical experiment, 0.1 g Pd/C catalyst (5 wt% Pd), 25 ml KBr aqueous solution (0.1 mol l^−1^) were added into the reactor. The reactor was sealed and purged with N_2_ to remove the air in the reactor. After stirring for 1 h at 30 °C, the solid Pd/C catalyst was recovered by filtration, followed by washing using ethanol aqueous solution of 20 v% ethanol (5 × 10 ml), and then was washed with 10 ml water. Finally, the Pd/C catalyst recovered by filtration was dried at 100 °C in a vacuum oven for 5 h, and the modified catalyst is named as m-Pd/C.

The catalysts were characterized by X-ray diffraction, X-ray photoelectron spectroscopy (XPS), transmission electron microscopy (TEM), and high-resolution TEM (HRTEM), inductively Coupled Plasma-Atomic Emission Spectroscopy (ICP-AES) techniques. X-ray diffraction measurements were conducted on an X-ray diffractometer (D/MAX-RC, Japan) operated at 40 kV and 200 mA with Cu Kα (λ=0.154 nm) radiation. TEM and HRTEM images were measured on a JEOL-2011F electron microscope operating at 200 kV. Before measurement, the catalyst was ground, suspended in ethanol, and dispersed by ultrasonic treatment. The obtained dispersion was transferred to a copper-grid-supported carbon film. The XPS measurements were carried out on an ESCAL Lab 220i-XL spectrometer at a pressure of ∼3 × 10^−9^ mbar (1 mbar=100 Pa) using Al Kα as the excitation source (*hν*=1486.6 eV) and operated at 15 kV and 20 mA. The contents of bromide anions adsorbed on the m-Pd/C catalysts were determined by ICP.

### Reaction

The reaction was carried out in a Teflon-lined stainless-steel reactor of 20 ml with a magnetic stirrer. The reactor was connected to a hydrogen cylinder of the reaction pressure, so that hydrogen of fixed pressure could be supplied continuously. The pressure was determined by a pressure transducer (FOXBORO/ICT, Model 93), which could be accurate to ±0.025 MPa. In a typical experiment, suitable amount of reactant, catalyst, solvent and salt (if used) were loaded into the reactor. The reactor was sealed and purged with hydrogen to remove the air at room temperature. Then the reactor was placed in a furnace at desired temperature. Hydrogen was introduced into the reactor and the stirrer was started with a stirring speed of 800 r.p.m. After the reaction, the reactor was placed in ice water and the gas was released and collected in a gas bag that was purged with H_2_ five times. The gaseous sample was analysed using a GC (Agilent 4890D) equipped with a TCD detector and a packed column (Carbon molecular sieve TDX-01, 1 m in length and 3 mm in diameter) using Argon as the carry gas. A known amount of internal standard (biphenyl) was added into the reactor. The liquid reaction mixture in the reactor was transferred into a centrifuge tube. The reactor was washed using ethyl acetate, which was combined the reaction mixture. The catalyst was separated by centrifugation. The quantitative analysis of the liquid products was conducted using a GC (Agilent 6820) equipped with a flame ionization detector (FID) and a HP-5MS capillary column (0.25 mm in diameter, 30 m in length). Identification of the products and reactant was done using a GC–MS (Agilent 5977A, HP-5MS capillary column (0.25 mm in diameter, 30 m in length)) as well as by comparing the retention time with respective standards in GC traces. The conversion of anisole and selectivities of the products were calculated from the GC data.

### Recycling of the catalyst

The reusability of m-Pd/C catalyst was tested for anisole transformation in H_2_O/CH_2_Cl_2_ system. After the reaction, the reaction mixture was centrifuged and the solid m-Pd/C catalyst was recovered by filtration, followed by washing using ethanol aqueous solution of 20 v% ethanol (5 × 10 ml), and then was washed with 10 ml water. The m-Pd/C catalyst was reused directly for the next run after drying at 100 °C for 5 h in a vacuum oven. To further check the stability of the catalyst, the m-Pd/C catalyst in H_2_O/CH_2_Cl_2_ was stirred at 120 °C for 40 h without anisole, which was equivalent to 8 cycles. After the stirring, the mixture was centrifuged and the solid m-Pd/C catalyst was recovered by filtration, followed by washing using ethanol aqueous solution of 20 v% ethanol (5 × 10 ml), and then was washed with 10 ml water. The m-Pd/C catalyst was reused for anisole transformation in H_2_O/CH_2_Cl_2_ system after drying at 100 °C for 5 h in a vacuum oven.

### ^18^O isotope labelling of cyclohexanone

The reactor used was the same as that described above. In the experiment, 1.5 mmol anisole, 0.03 g m-Pd/C, 0.2 ml H_2_^18^O and 7.8 ml CH_2_Cl_2_ were loaded into the reactor. The reactor was sealed and purged with hydrogen to remove the air at room temperature. Then the reactor was placed in a furnace at 90 °C. 2 MPa H_2_ was introduced into the reactor and the stirrer was started with a stirring speed of 800 r.p.m. After the reaction, the reactor was placed in ice water and the gas was released. The reaction mixture was transferred into a centrifuge tube and the catalyst was separated by centrifugation. Identification of the ^18^O-cyclohexanone was detected using a GC–MS (Agilent 5977A).

### Computational details

DFT calculations were performed with the Vienna *Ab initio* Simulations Package (VASP) code^39^. The electron exchange and correlation energy were modified within the generalized gradient approximation (GGA) in the Perdew-Burke-Ernzerhof formalism (PBE)^40^. Core electron interactions were described with the projector- augmented wave (PAW) method^40^. The methods used have been applied to DFT studies of arene derivatives and surface sites of Pd nanoparticles^41^.

In this work, the slab Pd (111) surface was used to represent the surface of Pd nanoparticles. Four layers (4 × 4) Pd (111) was used, in which the first two layers with adsorbates were relaxed, and the two bottom layers were fixed during the optimization and the vacuum layer of Pd (111) was 15 Å. The Brillouin zone integration was employed using a Monkhorst-Pack grid with 4 × 4 × 1 for the slab Pd (111)^42^. The cutoff energy for the plane waves was set up to 400 eV and the smearing width was 0.2 eV. Force convergence was set to be lower than 0.02 eV/Å, and total energy convergence was set to be less than 10^−4^ eV.

The adsorption energy of molecule on the slab Pd (111) surface was calculated as follows:


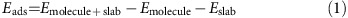


where *E*_ads_, *E*_molecule+slab_, *E*_molecule_ and *E*_slab_ represents the adsorption energy, the total energy after molecule is adsorbed on the slab Pd (111) surface, the energy of isolated molecule, and the total energy of the slab, respectively^41^.

### Data availability

All relevant data are available from the authors on reasonable request.

## Additional information

**How to cite this article:** Meng, Q. *et al*. Synthesis of ketones from biomass-derived feedstock. *Nat. Commun.* **8,** 14190 doi: 10.1038/ncomms14190 (2017).

**Publisher's note**: Springer Nature remains neutral with regard to jurisdictional claims in published maps and institutional affiliations.

## Supplementary Material

Supplementary Information Supplementary Figures, Supplementary Table and Supplementary References.

## Figures and Tables

**Figure 1:**
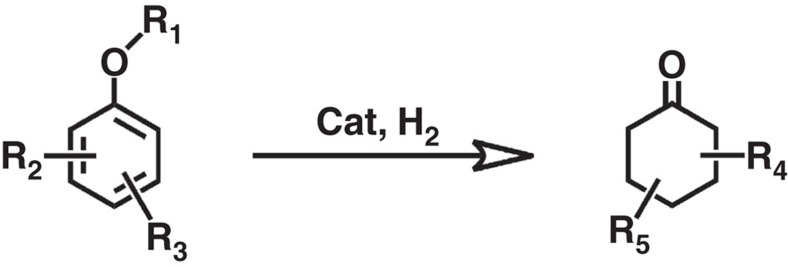
A sustainable route to produce ketones from aromatic ethers. Transformation of aromatic ethers with different substitute groups to corresponding ketones. R1, R2, R3, R4 and R5 stand for corresponding substitute groups.

**Table 1:**
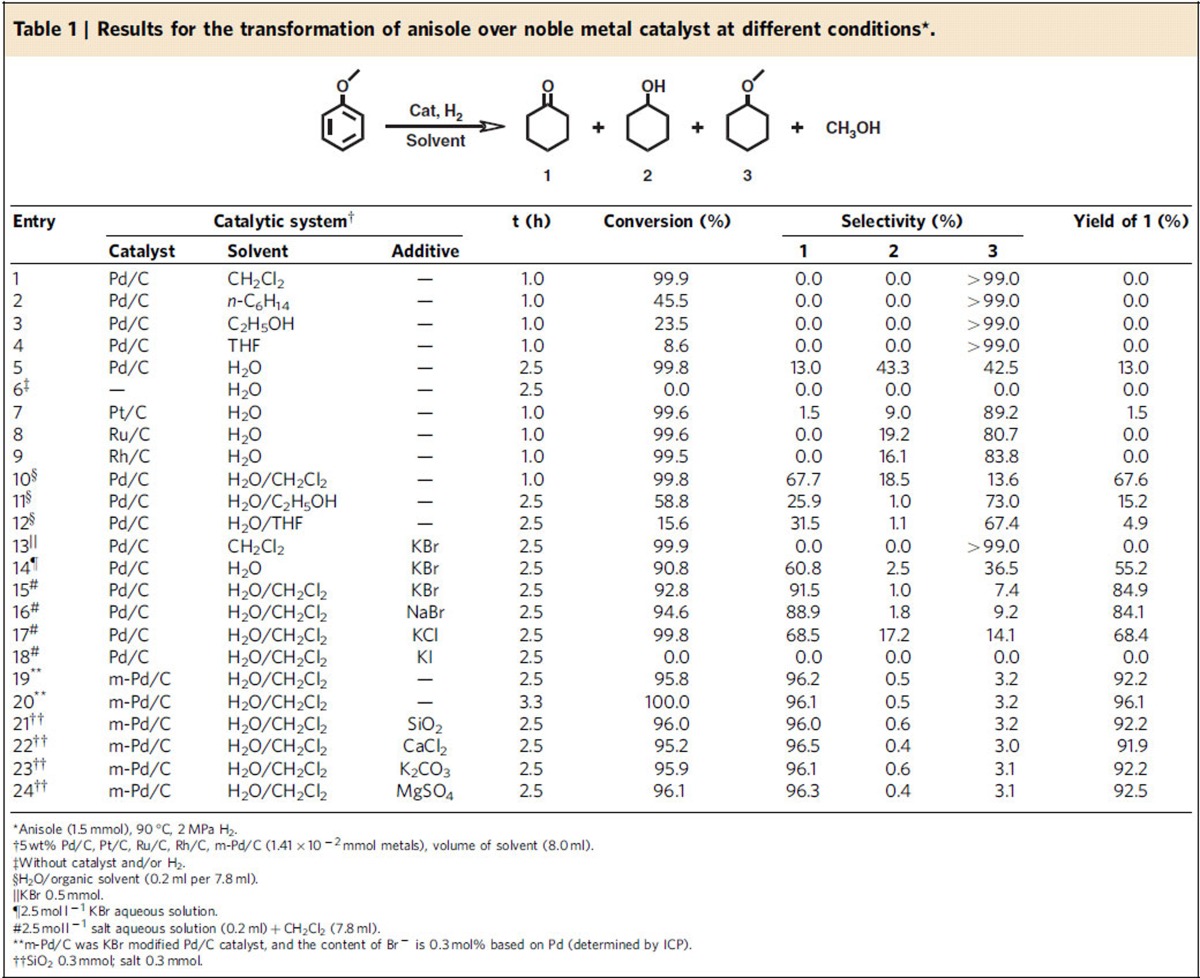
Results for the transformation of anisole over noble metal catalyst at different conditions^*^.

*Reaction conditions:**1**/LiHMDS/**2**/[Pd(*η*^3^-C_3_H_5_)Cl]_2_/S-IPr·HCl=200/200/100/2.5/5; 0.1 M of ketone **1**; T=30^o^C; B/L and *dr* was determined by ^1^H NMR, *dr* is the ratio of (±)-(*syn,anti*)-**3**/other diastereoisomers; Isolated yield. †T=50 ^o^C. ‡Solvent=THF. §OBoc of **2** was replaced with OP(OEt)_2_. ||The yield was determined by ^1^H NMR.

**Table 2:**
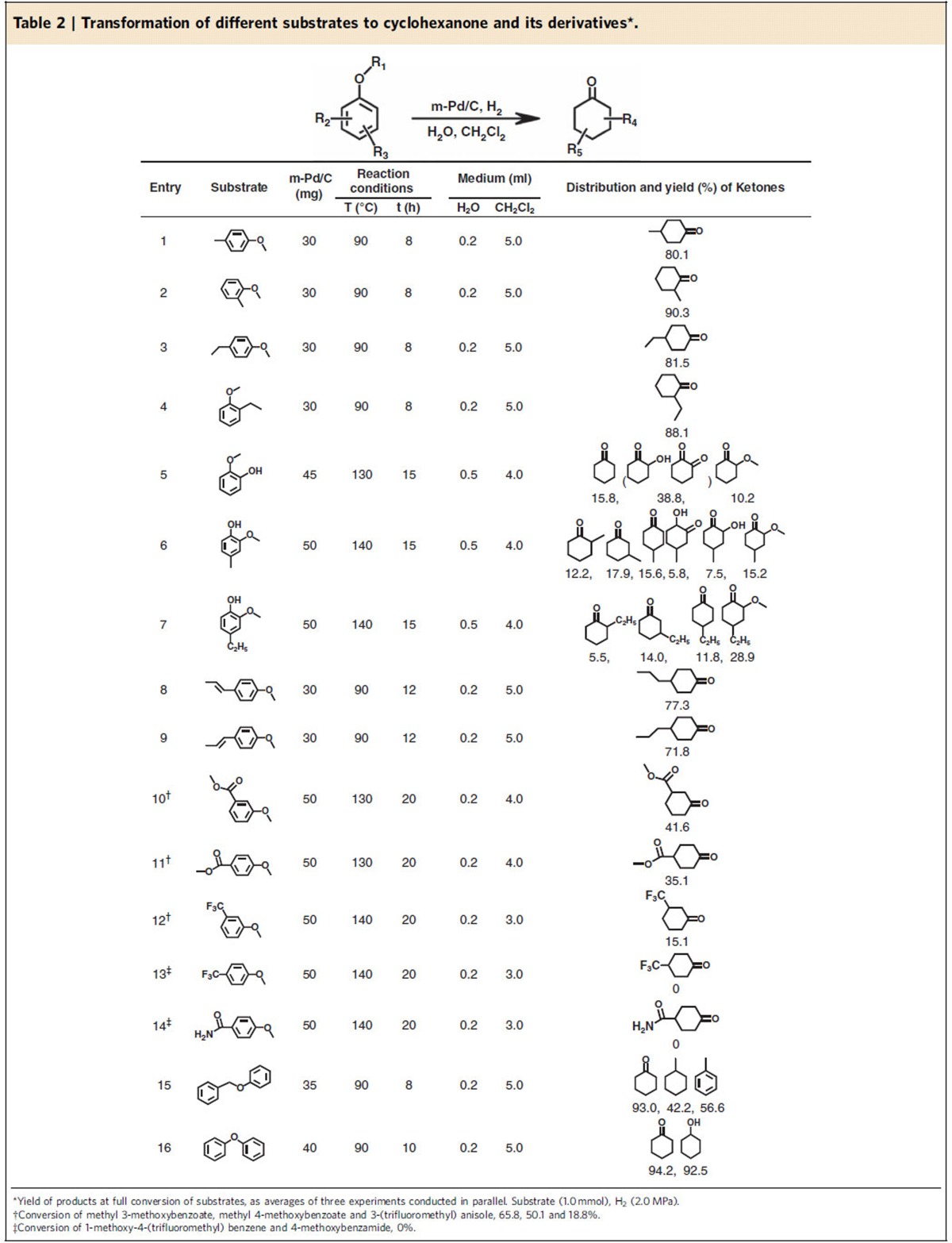
Transformation of different substrates to cyclohexanone and its derivatives^*^.

*Reaction conditions:**1**/LiHMDS/**2**/[Pd(*η*^3^-C_3_H_5_)Cl]_2_/S-IPr·HCl=200/200/100/2.5/5; 0.1 M of ketone **1**; T=30^o^C; B/L and *dr* was determined by ^1^H NMR, *dr* is the ratio of (±)-(*syn,anti*)-**3**/other diastereoisomers; Isolated yield. †T=50 ^o^C. ‡Solvent=THF. §OBoc of **2** was replaced with OP(OEt)_2_. ||The yield was determined by ^1^H NMR.
