# Supplementary material for: Synthesis of ketones from biomass-derived feedstock
Source: Nat Commun. 2017 Jan 31;8:14190. doi: 10.1038/ncomms14190 (PMC5290317; doi:10.1038/ncomms14190)
Supplement: Supplementary Information — Supplementary Figures, Supplementary Table and Supplementary References. [file ncomms14190-s1.pdf]

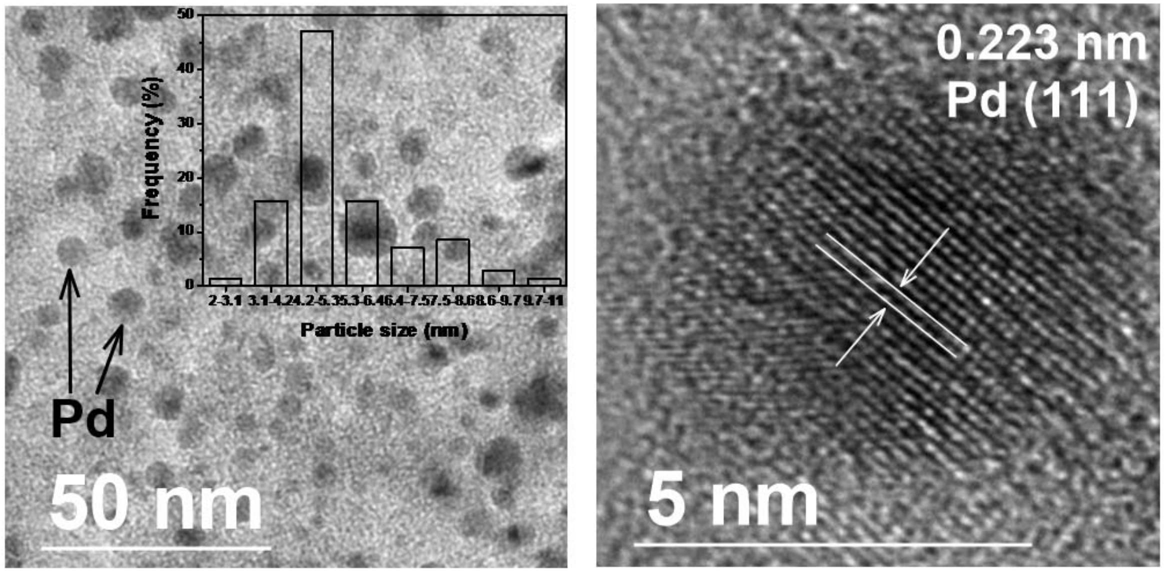

Supplementary Figure 1. TEM, HRTEM images, and Pd particle size distribution of the Pd/C catalyst.

Notes: Supplementary Fig. 1 shows the TEM, HRTEM images, and Pd particle size distribution of the Pd/C catalyst. The particles size of the Pd was mainly in the range of 3-6 nm, and the crystalline nature of the Pd nanoparticles can be observed[1, 2].

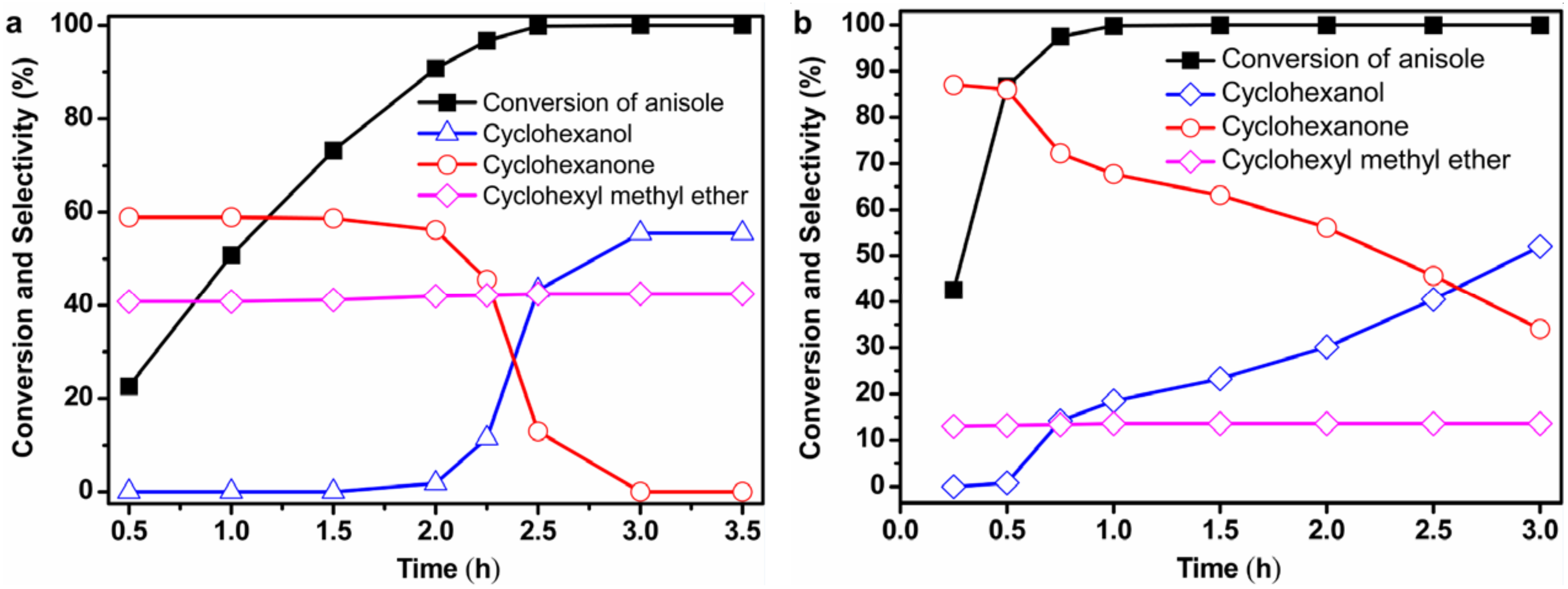
a
b
Conversion and Selectivity (%)
Time (h)
Conversion of anisole
Cyclohexanol
Cyclohexanone
Cyclohexyl methyl ether
0
20
40
60
80
100
0.5
1.0
1.5
2.0
2.5
3.0
3.5
10
30
50
70
90
0.0

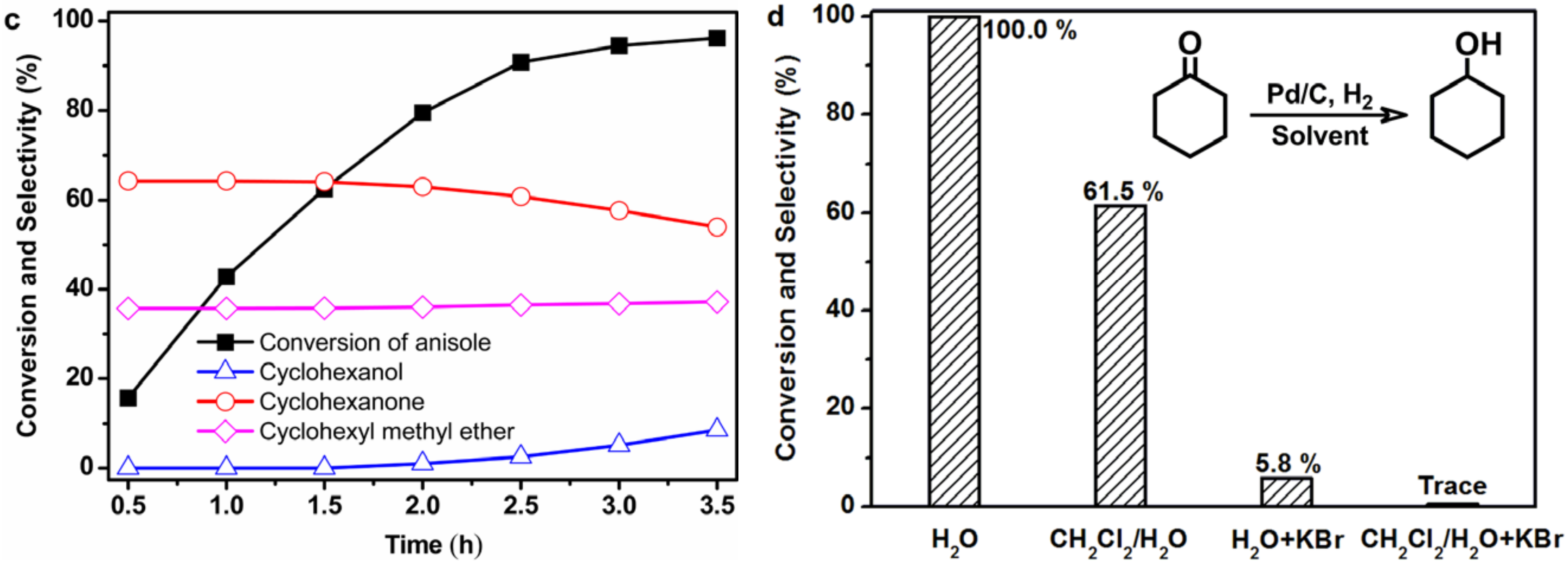

Supplementary Figure 2. Effect of reaction time on the conversion and product distribution of the reaction over Pd/C catalyst in neat H_2O (8.0 mL) (a), H_2O (0.2 mL) / CH_2Cl_2 (7.8 mL) (b), and 2.5 M KBr aqueous solution (8.0 mL) (c). Reaction conditions: anisole (1.5 mmol), 5 wt% Pd/C (0.03 g, 1.41×10^{-2} mmol Pd), 90 ºC, 2 MPa. (d) The effects of CH_2Cl_2 and/or KBr on the hydrogenation of cyclohexanone over Pd/C in water. Reaction conditions: cyclohexanone (1.5 mmol), 5 wt% Pd/C (0.03 g, 1.41×10^{-2} mmol Pd), 90 ºC, 2.5 h, 2 MPa; solvent: H_2O (8.0 mL); H_2O (0.2 mL) / CH_2Cl_2 (7.8 mL); KBr aqueous solution (2.5 M, 8.0 mL); KBr aqueous solution (2.5 M, 0.2 mL)/CH_2Cl_2 (7.8 mL).

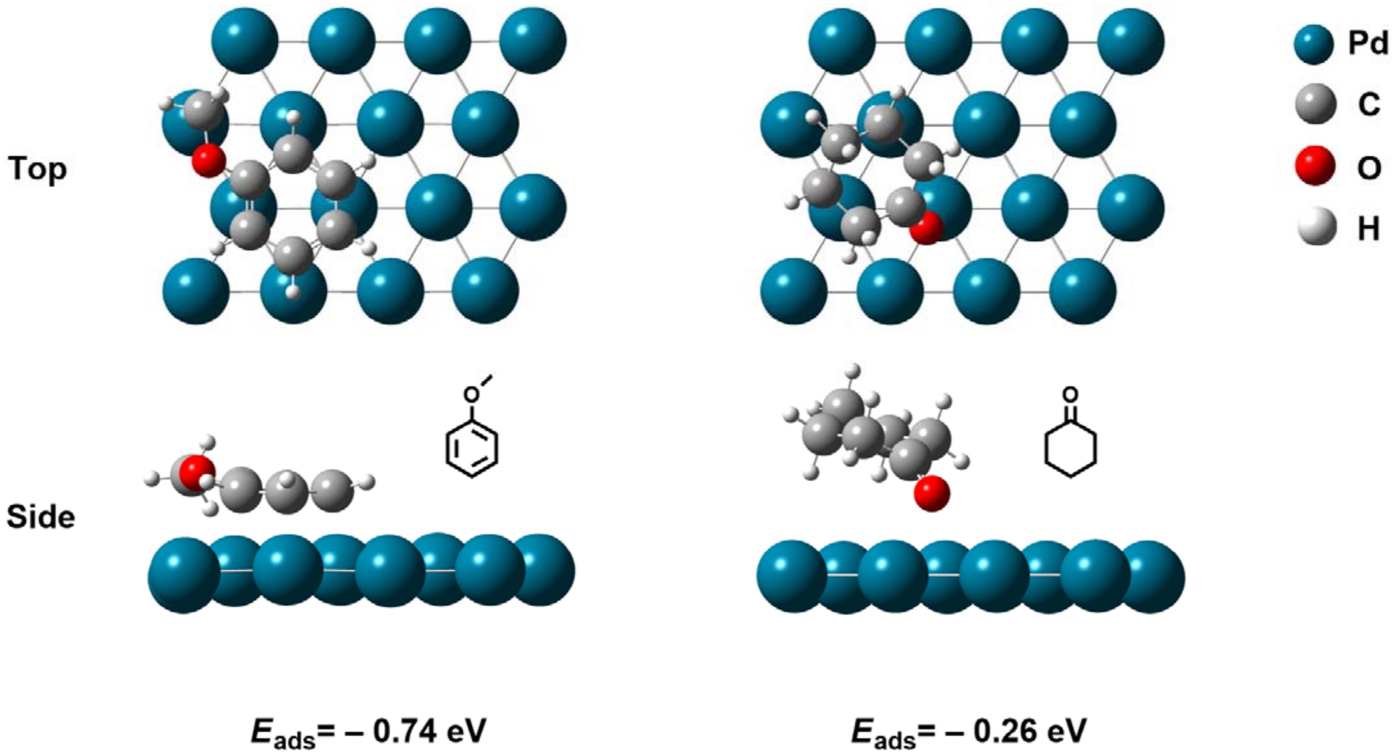

Supplementary Figure 3. Adsorption configurations of anisole and cyclohexanone on Pd (111) surface.

Notes: The transformation of anisole over Pd/C catalyst gave remarkably high selectivity of cyclohexanone in H_2O/CH_2Cl_2 in the presence of KBr. We carried out further experiments in order to explain this phenomenon, and the results are presented in Supplementary Fig. 2. Cyclohexanone, cyclohexanol and cyclohexyl methyl ether and methanol were the only reaction products observed in the present study. To explain this result, we calculated the adsorption energy of anisole and cyclohexanone on Pd (111) surface by DFT method[3-5], and the results are presented in Supplementary Fig. 3.

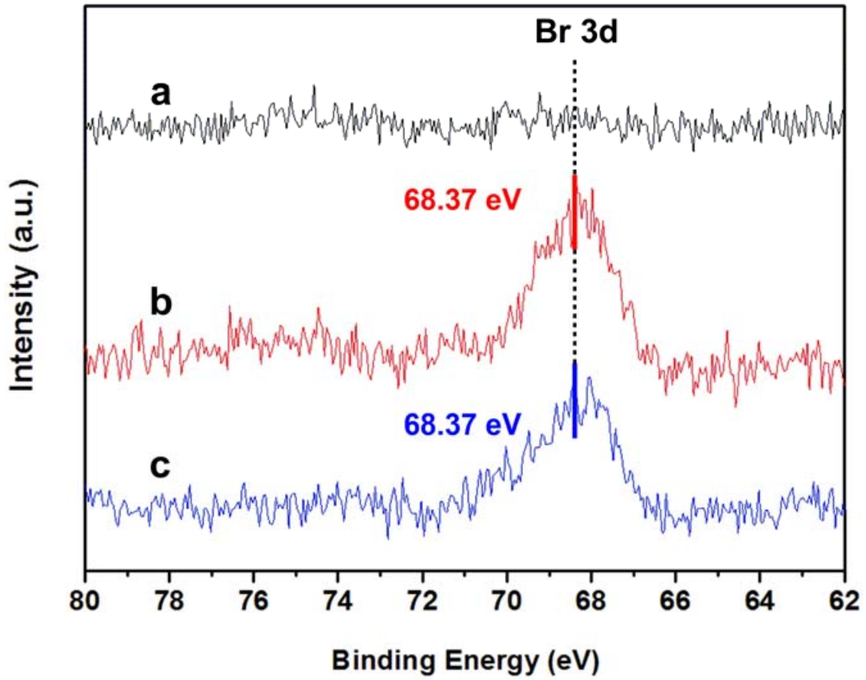

Supplementary Figure 4. XPS spectra of the Br 3d in the Pd/C catalyst (a), m-Pd/C catalyst (b) and m-Pd/C catalyst after 5 cycles (c).

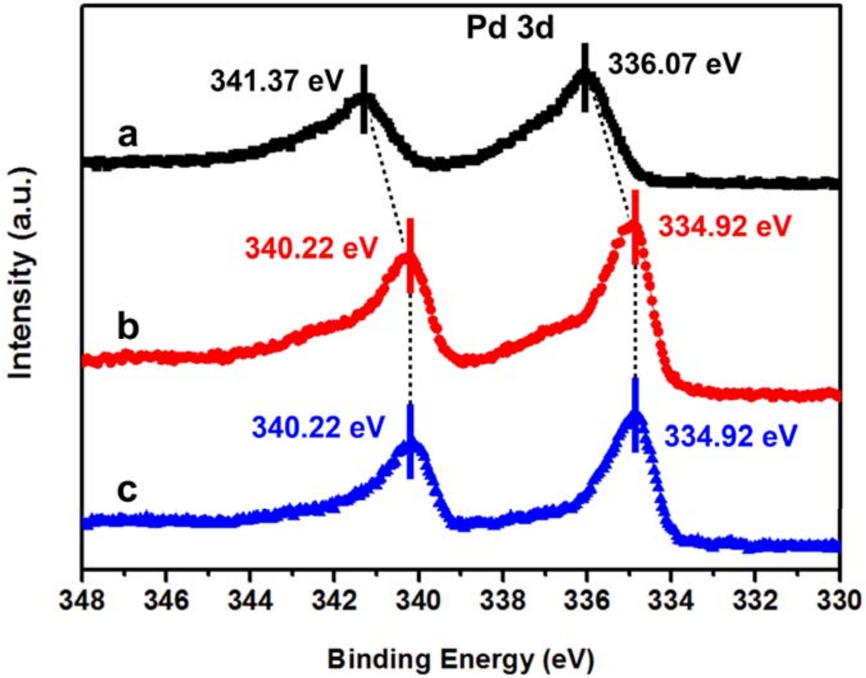

Supplementary Figure 5. XPS spectra of the Pd 3d in Pd/C catalyst (a), m-Pd/C catalyst (b) and m-Pd/C catalyst after 5 cycles (c).

Notes: The XPS method was used to study the Pd/C, m-Pd/C, and m-Pd/C after 5 cycles. The XPS spectra are given in Supplementary Figs. 4 and 5. The XPS peak at 68.37 eV corresponds to the Br $3d_{5/2}$ orbital, shown in the Supplementary Fig. 4b, confirmed that bromide anions were adsorbed on the surface of m-Pd/C catalyst, as compared with Pd/C catalyst (Supplementary Fig. 4a)[6, 7]. Supplementary Fig. 5 shows the XPS spectra of Pd 3d binding energy region of Pd/C (Supplementary Fig. 5a) and m-Pd/C (Supplementary Fig. 5b) catalysts. It can be seen that two peaks appeared at 341.37 eV and 336.07 eV which are related to Pd^0 $3d_{3/2}$ and Pd^0 $3d_{5/2}$ shifted to lower binding energies (340.22 eV and 334.92 eV) after the pretreatment by KBr, confirming the strong interaction between Pd species and Br anions[8-9]. The figures also show that the interaction between Br anions and Pd did not change after 5 cycles.

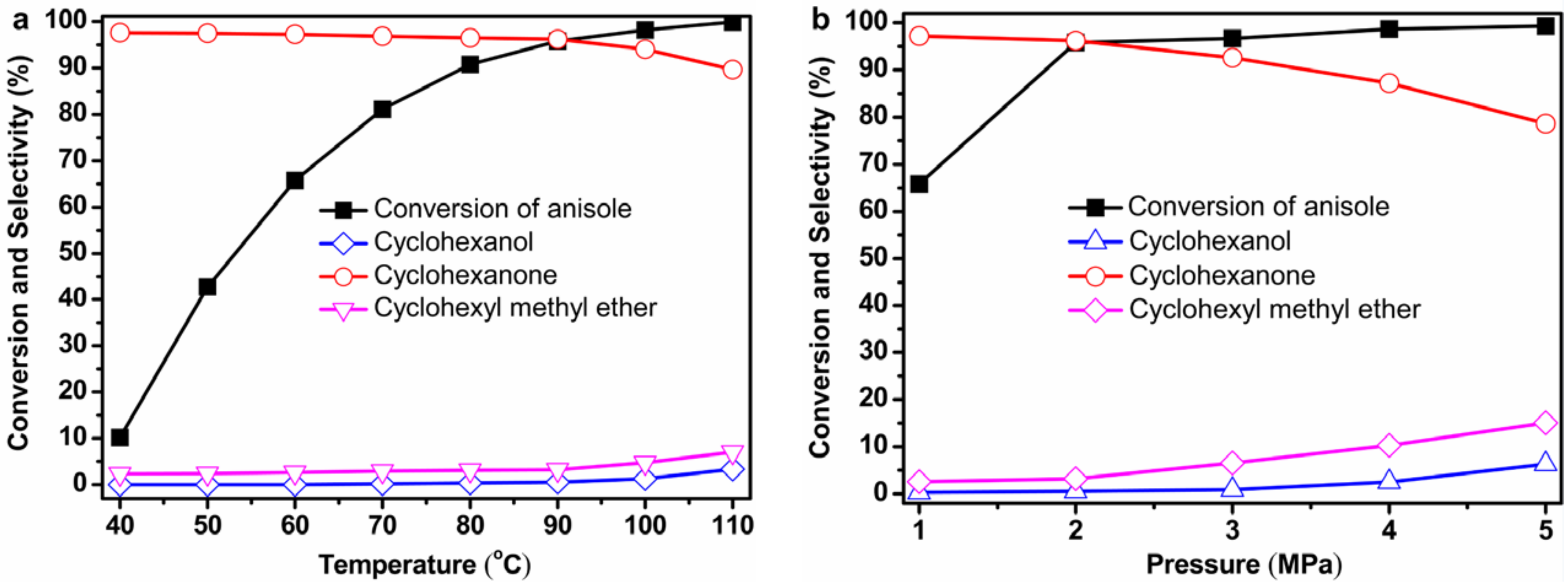
a
b
Conversion and Selectivity (%)
Temperature (°C)
Pressure (MPa)
Conversion of anisole
Cyclohexanol
Cyclohexanone
Cyclohexyl methyl ether

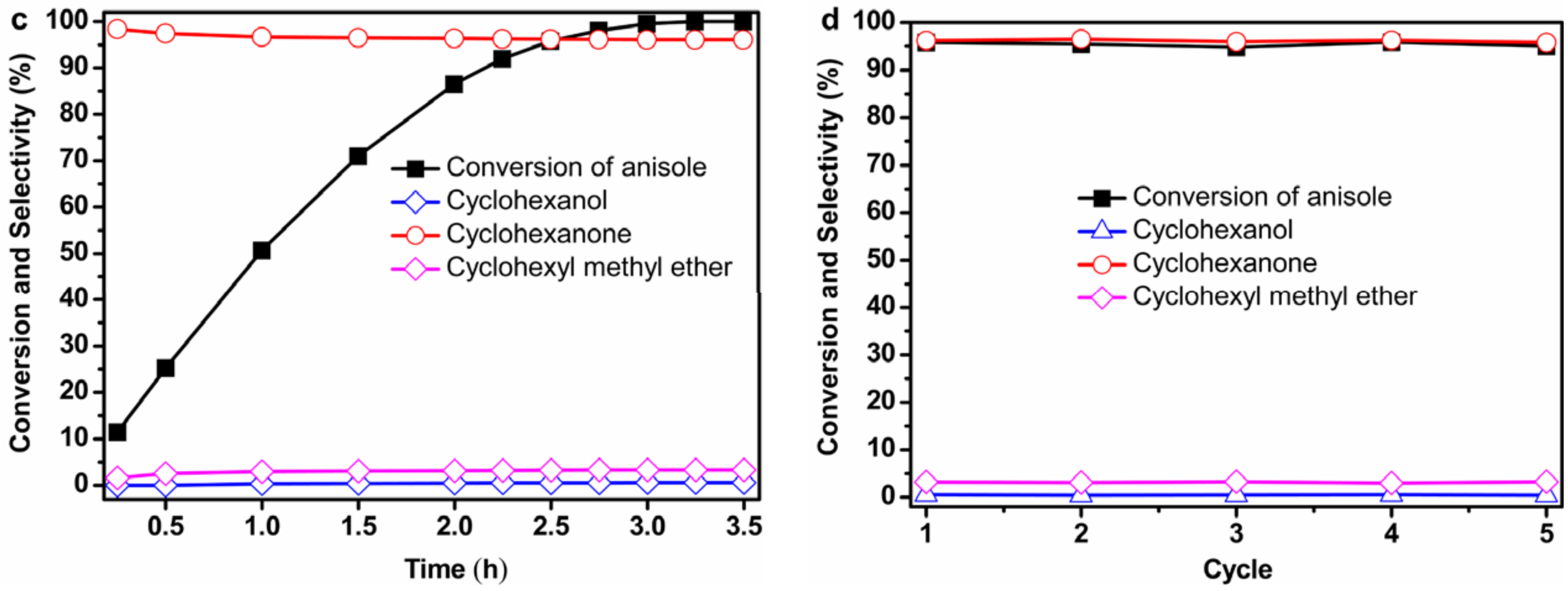

Supplementary Figure 6. Effects of temperature (a), H_2 pressure (b), and reaction time (c) on the conversion and product distribution of the reaction over m-Pd/C catalyst at fixed reaction volume and content of H_2O, and reusability of the m-Pd/C catalyst (d). Reaction conditions: anisole (1.5 mmol), 5 wt% m-Pd/C (0.03 g, 1.41×10^{-2} mmol Pd), water 0.2 mL, CH_2Cl_2 7.8 mL. (a) 2.5 h, 2 MPa; (b) 90 ºC, 2.5 h; (c) 90 ºC, 2 MPa; (d) 90 ºC, 2.5 h, 2 MPa.

Notes: The effect of temperature, H_2 pressure, and time on the conversion and product distribution of the reaction over the m-Pd/C catalyst in H_2O/CH_2Cl_2, stability and regenerability of the m-Pd/C catalyst are shown in Supplementary Fig. 6. Supplementary Fig. 6a indicates that the selectivity of cyclohexanone was higher than 96 % at lower temperature (<90 ºC). However, the selectivity to cyclohexyl methyl ether and cyclohexanol increased with temperature after 90 ºC. The effect of H_2 pressure on the reaction was studied with a time of 2.5 h (Supplementary Fig. 6b), at which the anisole could not be converted completely so that the effect of pressure can be shown clearly. As expected, the conversion of anisole increased with H_2 pressure, but the selectivity to cyclohexanone decreased considerably after 2 MPa, and the selectivity of cyclohexyl methyl ether increased obviously. The influence of time on the reaction at 90 ºC and 2 MPa is illustrated in Supplementary Fig. 6c. Nearly of all the anisole could be converted at the reaction time of 3.3 h, and selectivity to cyclohexanone was as high as 96.1%. Thus a yield of cyclohexanone

could approach 96.0% in the catalytic system. The conversion of anisole and selectivity to cyclohexanone did not change notably after the catalyst was reused five times (Supplementary Fig. 6d).

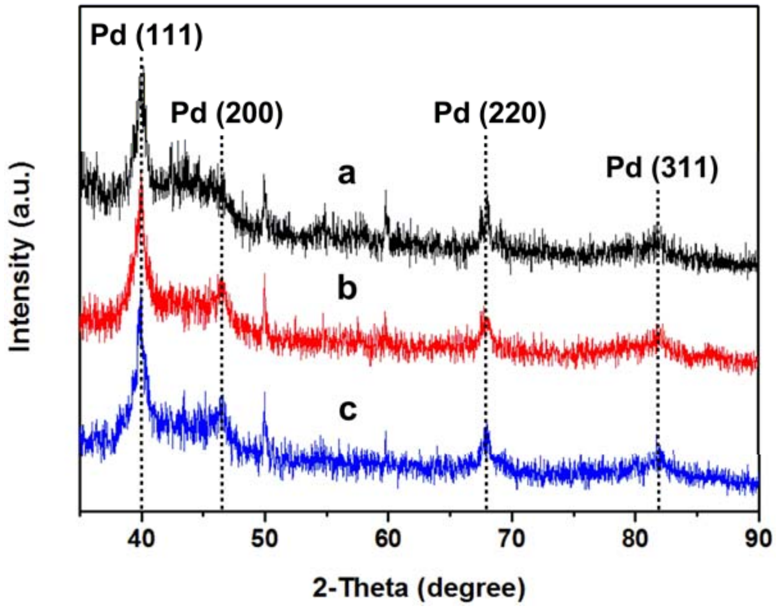

Supplementary Figure 7. XRD patterns of Pd/C catalyst (a), m-Pd/C catalyst (b) and m-Pd/C catalyst after 5 cycles (c).

Notes: Supplementary Fig. 7 exhibits the XRD patterns of Pd/C, m-Pd/C catalyst and the m-Pd/C catalyst after 5 cycles. It is shown that the reflection for the m-Pd/C catalyst (Supplementary Fig. 7b) and the m-Pd/C catalyst after 5 cycles (Supplementary Fig. 7c) are exactly the same as that of Pd/C catalyst (Supplementary Fig. 7a). The peaks appeared at around 40 °, 47 °, 68 °, and 82 ° are ascribed respectively to (111), (200), (220) and (311) lattice planes, a typical signature for Pd with a face-centered cubic (fcc) lattice[10, 11]. The XRD patterns suggests that the crystalline of metallic Pd remained after the pretreatment and reaction.

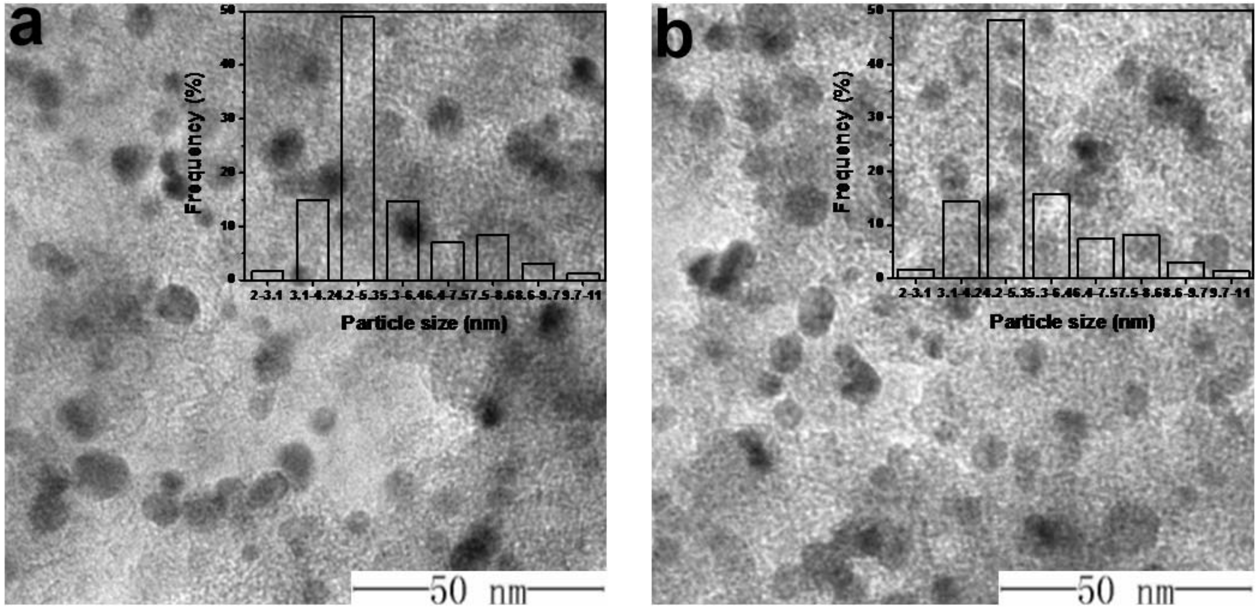

Supplementary Figure 8. TEM images and Pd particle size distributions of m-Pd/C catalyst (a) and m-Pd/C catalyst after 5 cycles (b).

Notes: The TEM images and Pd particle size distributions of the m-Pd/C and the m-Pd/C after five cycles are shown in Supplementary Fig. 8. It can be known from the images that the particle size of the Pd particles in the m-Pd/C and the m-Pd/C after five cycles were nearly the same. The Pd nanoparticles in the m-Pd/C catalysts are mainly in the range of 3-6 nm.

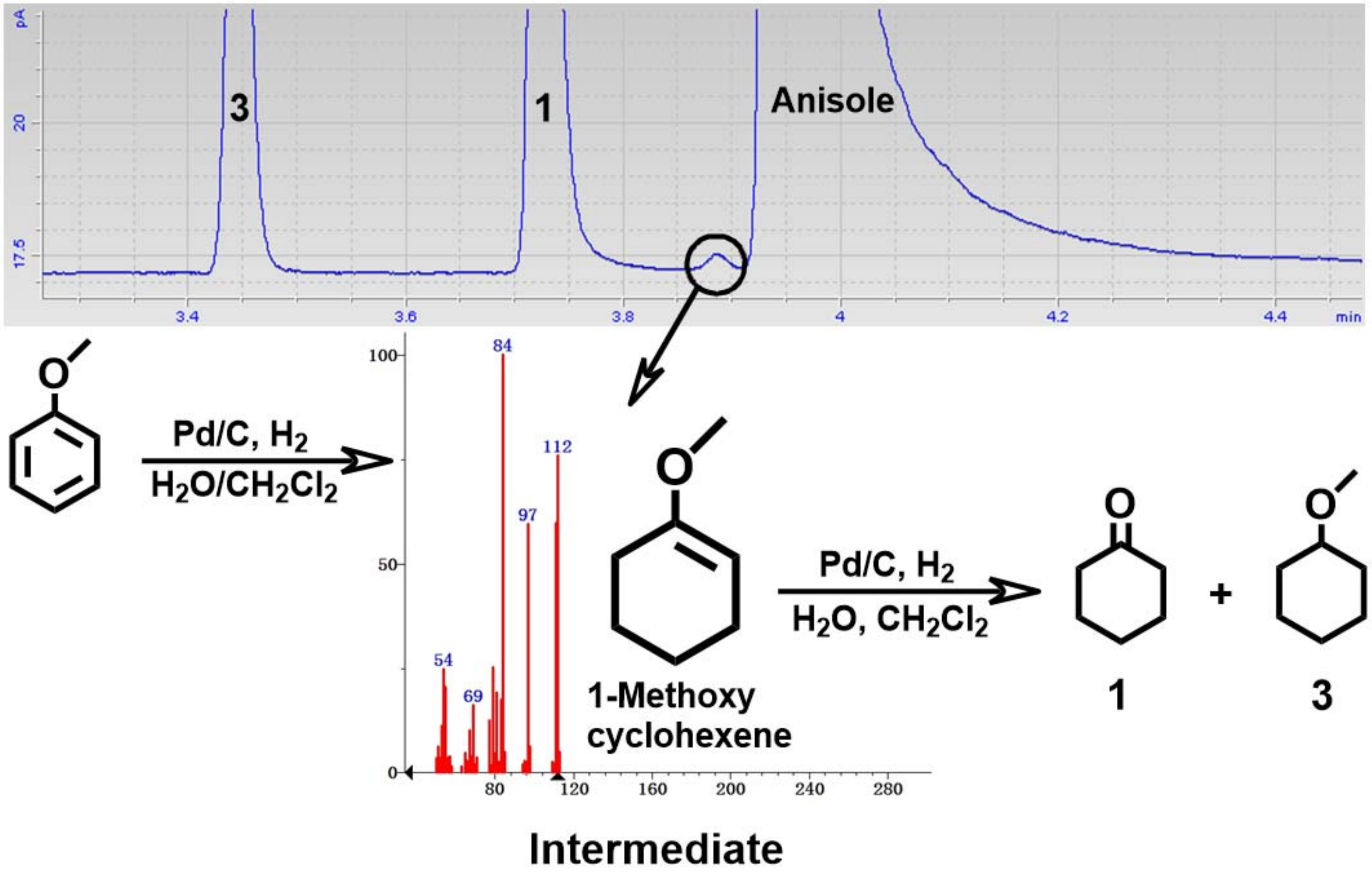

Supplementary Figure 9. Identification of the intermediate in the transformation of anisole.

Reaction conditions: anisole (1.5 mmol), Pd/C (5 wt% Pd, 0.03 g), H_2O (0.2 mL), CH_2Cl_2 (7.8 mL), 30 ºC; 0.3 MPa H_2, 1.0 h, 800 rpm.

Notes: The identification of the intermediate in the transformation of anisole was carried out in a Teflon-lined stainless-steel reactor of 20 mL with a magnetic stirrer. The reactor was connected to a hydrogen cylinder of the reaction pressure, so that hydrogen of fixed pressure could be supplied continuously. The pressure was

determined by a pressure transducer (FOXBORO/ICT, Model 93), which could be accurate to ±0.025 MPa. In this experiment, 1.5 mmol anisole, 0.03 g Pd/C, 0.2 mL H_2O and 7.8 mL CH_2Cl_2 were loaded into the reactor. The reactor was sealed and purged with hydrogen to remove the air at room temperature. Then the reactor was placed in a furnace at 30 ºC. 0.3 MPa H_2 was introduced into the reactor and the stirrer was started with a stirring speed of 800 rpm. After 1 hour, the reactor was placed in a bath of liquid nitrogen very quickly and the gas was released immediately. After the refrigeration, the mixture was transferred into a centrifuge tube and the catalyst was separated by centrifugation. Identification of the intermediate was conducted using a GC-MS (Agilent 5977A) as well as by comparing the retention time to respective standards in GC traces.

As shown in Supplementary Fig. 9, the intermediate 1-methoxycyclohexene was detected in the transformation of anisole. In our experiments at higher reaction temperature (90 ºC) and H_2 pressure (2 MPa), the intermediate could not be detected because the hydrolysis rate of 1-methoxycyclohexene was much faster than the hydrogenation of anisole to 1-methoxycyclohexene.

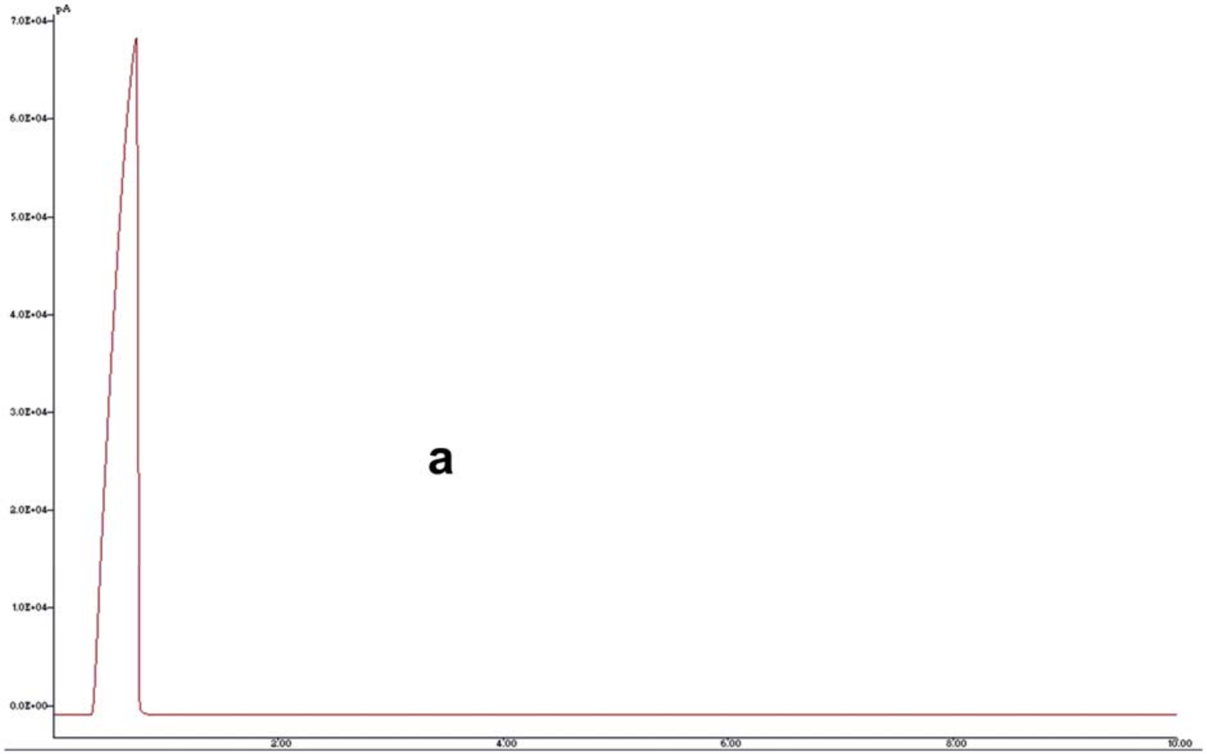
pA
7.0E+04
6.0E+04
5.0E+04
4.0E+04
3.0E+04
2.0E+04
1.0E+04
0.0E+00
2.00
4.00
6.00
8.00
10.00
a

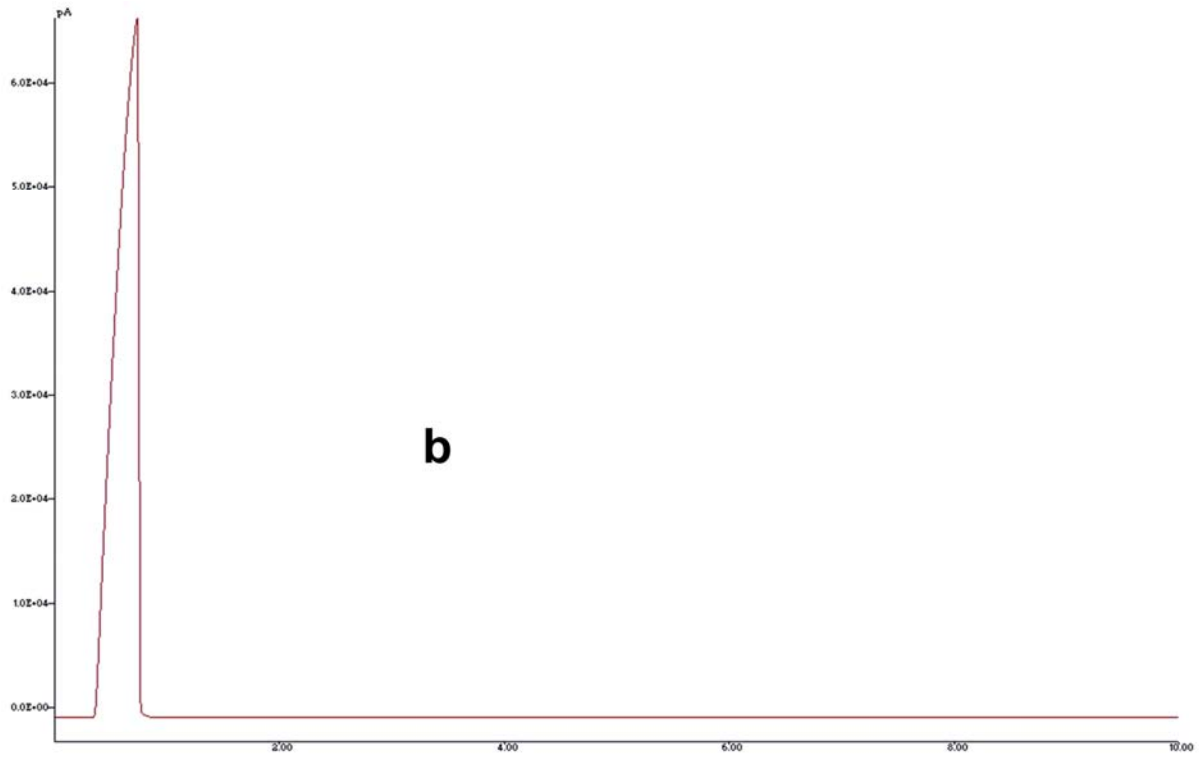

Supplementary Figure 10. GC traces of: (a) the gaseous sample obtained at 90 ^{o}C after reaction 2.5 h (Supplementary Table 1, entry 1. Other reaction conditions: anisole, 1.5 mmol; m-Pd/C (5 wt% Pd), 0.03 g; solvent: 0.2 mL H_2O and 7.8 mL CH_2Cl_2; H_2, 2 MPa); (b) the blank gaseous sample, which was obtained from the reactor containing 2 MPa of H_2, 0.2 mL H_2O and 7.8 mL CH_2Cl_2.

Notes: All the GC traces of the gaseous samples were similar. As an example, Supplementary Fig. 10 presents the GC trace of a typical gaseous sample. GC trace of a blank gaseous sample (the reactor contained only 2 MPa of hydrogen, 0.2 mL H_2O and 7.8 mL CH_2Cl_2) is also given in the figure. The two GC traces are the same in that only hydrogen was detected in the gaseous sample. This indicates that no gaseous product was produced in the anisole transformation.

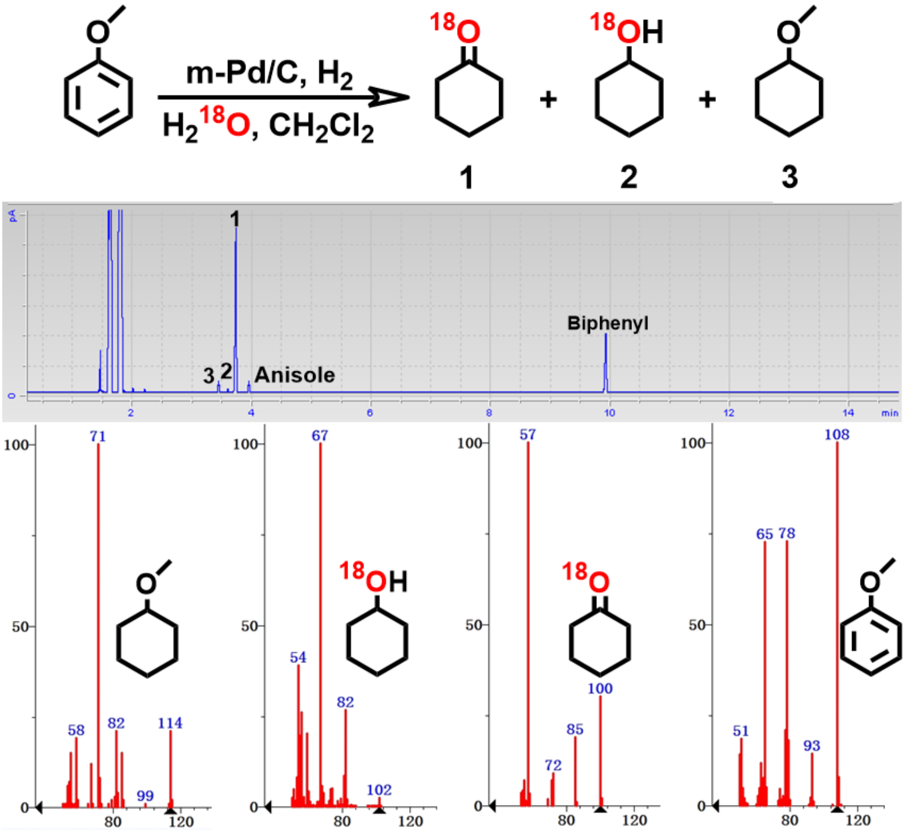

Supplementary Figure 11. MS spectra for ^{18}O labelling of cyclohexanone in the transformation of anisole over m-Pd/C catalyst.

Reaction conditions: anisole (1.5 mmol), m-Pd/C (5 wt% Pd, 0.03 g), $H_2{}^{18}O$ (0.2 mL), CH_2Cl_2 (7.8 mL), 90 ºC, 2 MPa H_2, 2.5 h, 800 rpm.

Notes: The transformation of anisole was carried out in the presence of 0.2 mL $H_2{}^{18}O$, and 7.8 mL CH_2Cl_2 at 90 ºC. After the reaction, the ^{18}O isotope abundance of the as-obtained products was measured by GC-MS, and the MS spectra for the ^{18}O labelling experiment is shown in Supplementary Fig. 11. It can be found that ^{18}O isotope appeared unambiguously in the C=^{18}O group of cyclohexanone[12, 13], which provides further evidence to support the for the reaction pathway.

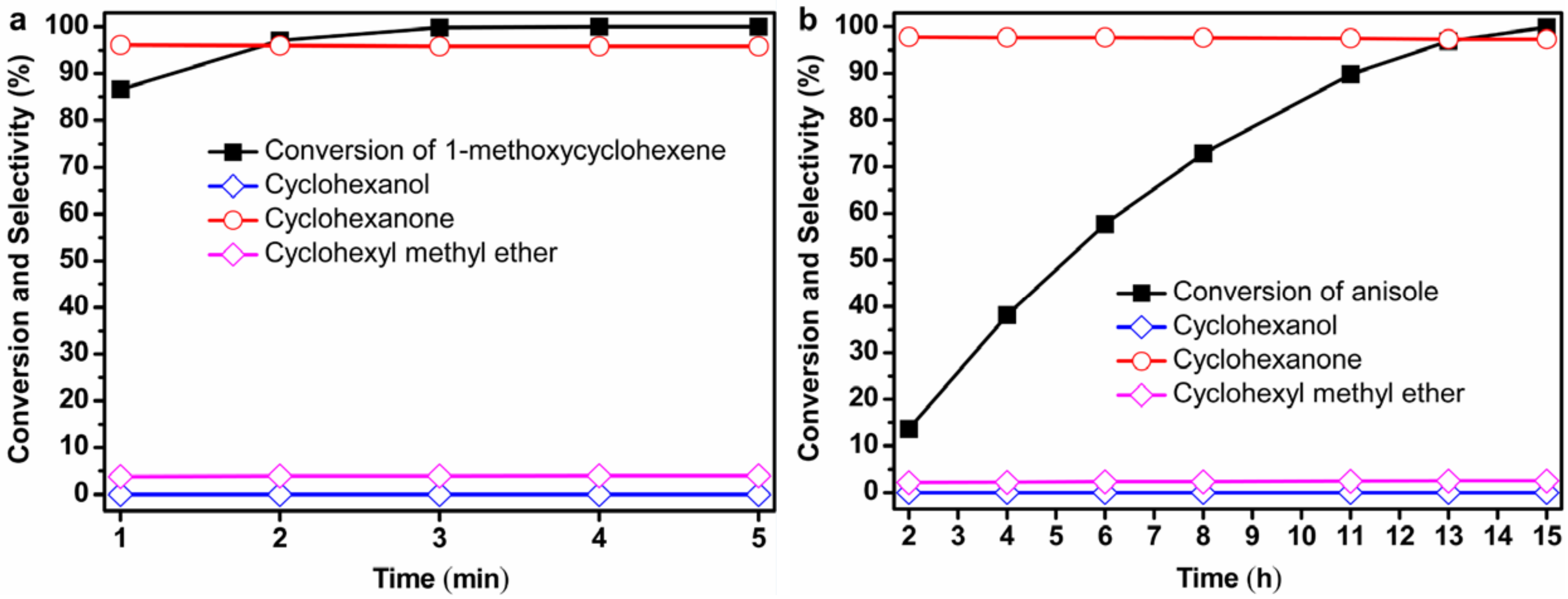

Supplementary Figure 12. Transformation of 1-methoxycyclohexene (a) and anisole (b) over m-Pd/C catalyst.

Reaction conditions: (a) 1-methoxycyclohexene (0.75 mmol), m-Pd/C (5 wt% Pd, 0.015 g), H_2O (0.1 mL), CH_2Cl_2 (3.9 mL), 30 ^oC, 2 MPa H_2, 800 rpm; (b) anisole (0.75 mmol), m-Pd/C (5 wt% Pd, 0.015 g), H_2O (0.1 mL), CH_2Cl_2 (3.9 mL), 40 ^oC, 2 MPa H_2, 800 rpm. The 1-methoxycyclohexene sample contained 50 mol% cyclohexanone dimethylacetal that converted into cyclohexanone completely[14], which has been considered when calculating the data.

Notes: To support the proposed pathway of cyclohexanone generation, we conducted the transformation of 1-methoxycyclohexene (Supplementary Fig. 12a). It was shown that 1-methoxycyclohexene was converted into cyclohexanone rapidly in the presence of m-Pd/C catalyst and H_2, and the selectivity to cyclohexanone could reach 95.8 % at complete conversion of 1-methoxycyclohexene. The selectivities of the products were similar to that of the transformation of anisole (Supplementary Fig. 12b), which further supports the proposed pathway.

Supplementary Table 1. Results for the transformation of anisole over m-Pd/C catalyst at different conditions. [a]

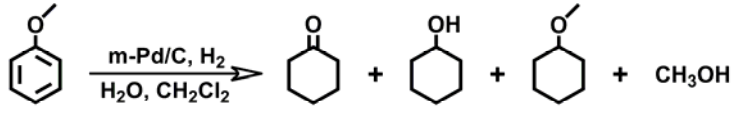

Entry	Catalytic system		t /T/ P (h/°C/MPa)	In (mmol)	Conversion (%)	Yield (%)			Yield of **1**+**2** (%)	Yield of **4** (%)
	Catalyst	Solvent				**1**	**2**	**3**		
1	m-Pd/C	H_2O/CH_2Cl_2	2.5/90/2	1.5	95.8	92.2	0.5	3.1	92.7	92.7
2	m-Pd/C	H_2O/CH_2Cl_2	3.25/90/2	1.5	100.0	96.1	0.5	3.2	96.6	96.5
3	m-Pd/C	H_2O/CH_2Cl_2	1.5/90/2	1.5	71.0	68.5	0.3	2.2	68.8	68.6
4	m-Pd/C	H_2O/CH_2Cl_2	2.5/80/2	1.5	91.6	88.4	0.3	2.8	88.7	88.3
5	m-Pd/C	H_2O/CH_2Cl_2	2.5/100/2	1.5	98.0	92.1	1.2	4.6	93.3	93.0
6	m-Pd/C	H_2O/CH_2Cl_2	2.5/90/1	1.5	65.8	64.0	0.2	1.6	64.2	64.3
7	m-Pd/C	H_2O/CH_2Cl_2	2.5/90/3	1.5	96.7	89.5	0.8	6.3	90.3	89.9

[a] Reaction conditions: anisole, 1.5 mmol; m-Pd/C (5 wt% Pd), 0.03 g, 0.2 mL H_2O, 7.8 mL CH_2Cl_2.

Notes: Results for the transformation of anisole over m-Pd/C catalyst at typical conditions are given in Supplementary Table 1. The yield of methanol is similar to that of the total yield of the cyclohexanone and cyclohexanol, indicating that cyclohexanone, cyclohexanol, methyl cyclohexyl ether and methanol were the only products derived from anisole.

Supplementary References

1. Liu, H. Z., Jiang, T., Han, B. X., Liang, S. G. & Zhou, Y. X. Selective phenol hydrogenation to cyclohexanone over a dual supported Pd-Lewis acid catalyst. *Science* **326**, 1250-1252 (2009).
2. Mazumder, V. & Sun, S. Oleylamine-mediated synthesis of Pd nanoparticles for catalytic formic acid oxidation. *J. Am. Chem. Soc.* **131**, 4588-4589 (2009).
3. Tan, Y. P., Khatua S., Jenkins, S. J., Yu, J. Q., Spencer, J. B. & King, D. A. Catalyst-induced changes in a substituted aromatic: A combined approach via experiment and theory. *Surf. Sci.* **589**, 173-183 (2005).
4. Rasmussen, A. M. H. & Hammer, B. Adsorption, mobility, and dimerization of benzaldehyde on Pt (111). *J. Chem. Phys.* **136**, 174706-174714 (2012).
5. Bonalumi, N., Vargas, A., Ferri, D. & Baiker, A. Theoretical and spectroscopic study of the effect of ring substitution on the adsorption of anisole on platinum. *J. Phys. Chem. B* **110**, 9956-9965 (2006).
6. Choudhary, V. R. & Samanta, C. Role of chloride or bromide anions and protons for promoting the selective oxidation of H_2 by O_2 to H_2O_2 over supported Pd catalysts in an aqueous medium. *J. Catal.* **238**, 28-38 (2006).
7. Peng, H. C., Xie, S. F., Park, J. H., Xia, X. H. & Xia, Y. N. Quantitative analysis of the coverage density of Br^- ions on Pd{100} facets and its role in controlling the shape of Pd nanocrystals. *J. Am. Chem. Soc.* **135**, 3780-3783 (2013).
8. Gong, Y. T., Zhang, P. F., Xu, X., Li, Y., Li, H. R. & Wang, Y. A novel catalyst Pd@ompg-C_3N_4 for highly chemoselective hydrogenation of quinoline under mild conditions. *J. Catal.* **297**, 272-280 (2013).
9. Wang, A. L., Xu, H., Feng, J. X., Ding, L. X., Tong, Y. X. & Li, G. R. Design of Pd/PANI/Pd sandwich-structured nanotube array catalysts with special shape effects and synergistic effects for ethanol electrooxidation. *J. Am. Chem. Soc.* **135**, 10703-10709 (2013).
10. Cao, M. N., Wu, D. S., Su, W. P. & Cao, R. Palladium nanocrystals stabilized by cucurbit [6] uril as efficient heterogeneous catalyst for direct C-H functionalization of polyfluoroarenes. *J. Catal.* **321**, 62-69 (2015).
11. Mondal, B., Acharyya, K., Howlader, P. & Mukherjee, P. S. Molecular cage impregnated palladium nanoparticles: efficient, additive-free heterogeneous catalysts for cyanation of aryl halides. *J. Am. Chem. Soc.* **138**, 1709-1716 (2016).
12. Zhang, M., Wang, Q., Chen, C. C., Zang, L., Ma, W. H. & Zhao, J. C. Oxygen atom transfer in the photocatalytic oxidation of alcohols by TiO_2: Oxygen isotope studies. *Angew. Chem. Int. Ed.* **48**, 6081-6084 (2009).
13. Meng, C., Yang, K., Fu, X. Z. & Yuan, R. S. Photocatalytic oxidation of benzyl alcohol by homogeneous $CuCl_2$/Solvent: a Model system to explore the role of molecular oxygen. *ACS Catal.* **5**, 3760-3766 (2015).

14. Procopio, A., Gaspari, M., Nardi, M., Oliverio, M., Tagarelli, A. & Sindona, G. Simple and efficient MW-assisted cleavage of acetals and ketals in pure water. *Tetrahedron Lett.* **48**, 8623-8627 (2007).
